# Experimentally Engineered Mutations in a Ubiquitin Hydrolase, UBP-1, Modulate *In Vivo* Susceptibility to Artemisinin and Chloroquine in Plasmodium berghei

**DOI:** 10.1128/AAC.02484-19

**Published:** 2020-06-23

**Authors:** Nelson V. Simwela, Katie R. Hughes, A. Brett Roberts, Michael T. Rennie, Michael P. Barrett, Andrew P. Waters

**Affiliations:** a Institute of Infection, Immunity and Inflammation, Wellcome Centre for Integrative Parasitology, University of Glasgow, Glasgow, Scotland, United Kingdom

**Keywords:** artemisinin, *Plasmodium berghei*, *Plasmodium falciparum*, drug resistance, malaria

## Abstract

As resistance to artemisinins (current frontline drugs in malaria treatment) emerges in Southeast Asia, there is an urgent need to identify the genetic determinants and understand the molecular mechanisms underpinning such resistance. Such insights could lead to prospective interventions to contain resistance and prevent the eventual spread to other regions where malaria is endemic. Reduced susceptibility to artemisinin in Southeast Asia has been primarily linked to mutations in the Plasmodium falciparum Kelch-13 gene, which is currently widely recognized as a molecular marker of artemisinin resistance. However, two mutations in a ubiquitin hydrolase, UBP-1, have been previously associated with reduced artemisinin susceptibility in a rodent model of malaria, and some cases of UBP-1 mutation variants associated with artemisinin treatment failure have been reported in Africa and SEA. In this study, we employed CRISPR-Cas9 genome editing and preemptive drug pressures to test these artemisinin susceptibility-associated mutations in UBP-1 in Plasmodium berghei sensitive lines *in vivo*. Using these approaches, we show that the V2721F UBP-1 mutation results in reduced artemisinin susceptibility, while the V2752F mutation results in resistance to chloroquine (CQ) and moderately impacts tolerance to artemisinins. Genetic reversal of the V2752F mutation restored chloroquine sensitivity in these mutant lines, whereas simultaneous introduction of both mutations could not be achieved and appears to be lethal. Interestingly, these mutations carry a detrimental growth defect, which would possibly explain their lack of expansion in natural infection settings. Our work provides independent experimental evidence on the role of UBP-1 in modulating parasite responses to artemisinin and chloroquine under *in vivo* conditions.

## Abstract (precis)

As resistance to artemisinins (current frontline drugs in malaria treatment) emerges in Southeast Asia, there is an urgent need to identify the genetic determinants and understand the molecular mechanisms underpinning such resistance. Such insights could lead to prospective interventions to contain resistance and prevent the eventual spread to other regions where malaria is endemic. Reduced susceptibility to artemisinin in Southeast Asia has been primarily linked to mutations in the Plasmodium falciparum Kelch-13 gene, which is currently widely recognized as a molecular marker of artemisinin resistance.

## INTRODUCTION

Artemisinins (ARTs) in artemisinin combinational therapies (ACTs) remain the mainstay of malaria treatment globally and thus far remain mostly effective in sub-Saharan Africa, where most of the disease burden occurs (1). However, ART (and even ACT) resistance has emerged in Southeast Asia (SEA), with a risk of spreading that is seriously threatening recent gains achieved in malaria control (2, 3). ART resistance is thought to be primarily conferred by specific mutations in the Plasmodium falciparum Kelch-13 (PfKelch13) gene, and such mutations are currently almost endemic in most parts of SEA (1, 4, 5). Phenotypically, these mutations are associated with delayed parasite clearance rates *in vivo* and with reduced susceptibility of ring stage parasites *in vitro* in ring stage survival assays (RSA) (3, 6). Interestingly, the prevalence of PfKelch13 mutations remains low outside SEA (7), and the few observed PfKelch13 polymorphisms in sub-Saharan Africa are not associated with treatment failure and/or delayed parasite clearance rates (8). Moreover, large-scale genome-wide association studies have revealed that polymorphisms in other genes such as multidrug resistance protein 2, ferredoxin, and others are also associated in SEA with delayed parasite clearance rates (9). More recently, mutations in an independent gene, P. falciparum coronin (PfCoronin), have been shown to confer enhanced survival in ring stage parasites exposed to dihydroartemisinin (DHA) (10). Deconvoluting the geographic complexities of ART resistance, genetic determinants, and the molecular mechanism involved would thus provide an avenue to contain or rescue emergent ART resistance through efficient surveillance and/or suitable combinational therapies.

Mutations in a ubiquitin hydrolase, UBP-1 (a close homologue to HAUSP or USP7), were previously identified to modulate susceptibility to ART and chloroquine (CQ) in the rodent-infectious malaria parasite Plasmodium chabaudi after sequential experimental evolution and selection with a series of antimalarial drugs (11). The reported drug-resistant phenotypes emerged from *in vivo* passage and exposure of the P. chabaudi drug-sensitive AS line to sublethal doses of pyrimethamine, CQ, mefloquine, and ARTs (11–13). Interestingly, in these P. chabaudi lineages, CQ resistance at 15 mg/kg emerged first, and from this uncloned line, whole-genome sequencing revealed two UBP-1 mutations (V2697F and V2728F) that were associated with the resistance phenotype (13, 14). Further selection of this uncloned CQ-resistant line generated lines with different drug resistance profiles, as follows: (i) a line resistant to 15 mg/kg mefloquine, (ii) a line resistant to CQ at 30 mg/kg, (iii) a line resistant to up to 300 mg/kg ART, which was selected from the CQ 30 mg/kg-resistant line, and (iv) a line resistant to up to 60 mg/kg artesunate. Upon further cloning and genome sequencing of these lines, it was found that the UBP-1 V2728F mutation was common in the ART-, CQ (30 mg/kg)-, and mefloquine-resistant lines, while the V2697F mutation only fixated upon artesunate selection (11, 12, 14). Due to the complexity of the selection procedure with multiple drugs, it has been difficult to confidently associate these UBP-1 mutations with ART and CQ susceptibility in the absence of appropriate reverse genetics approaches. Recently, these mutations have been introduced into UBP-1 in P. falciparum, and the V2721F equivalent has been shown to associate with increased DHA RSA survival with no CQ resistance phenotype, whereas the V2728F orthologue appeared to have no ART or CQ resistance profiles (15). More interestingly, UBP-1 mutation variants have been associated with decreased effectiveness of ARTs in Africa and some parts of Asia (16–19).

In our present study, we successfully engineered UBP-1 candidate mutations in an independent rodent model of P. berghei infection using a CRISPR-Cas9 genome editing system. We provide a causal link to the reduced ART and CQ susceptibility profiles of these mutant lines both *in vitro* and *in vivo*. We have also characterized their relative fitness compared to that of the wild-type nonmutant parasite.

## RESULTS

### CRISPR-Cas9-engineered mutations in UBP-1 confer *in vivo* selective advantage to ART and CQ pressure in Plasmodium berghei.

**FIG 1:**
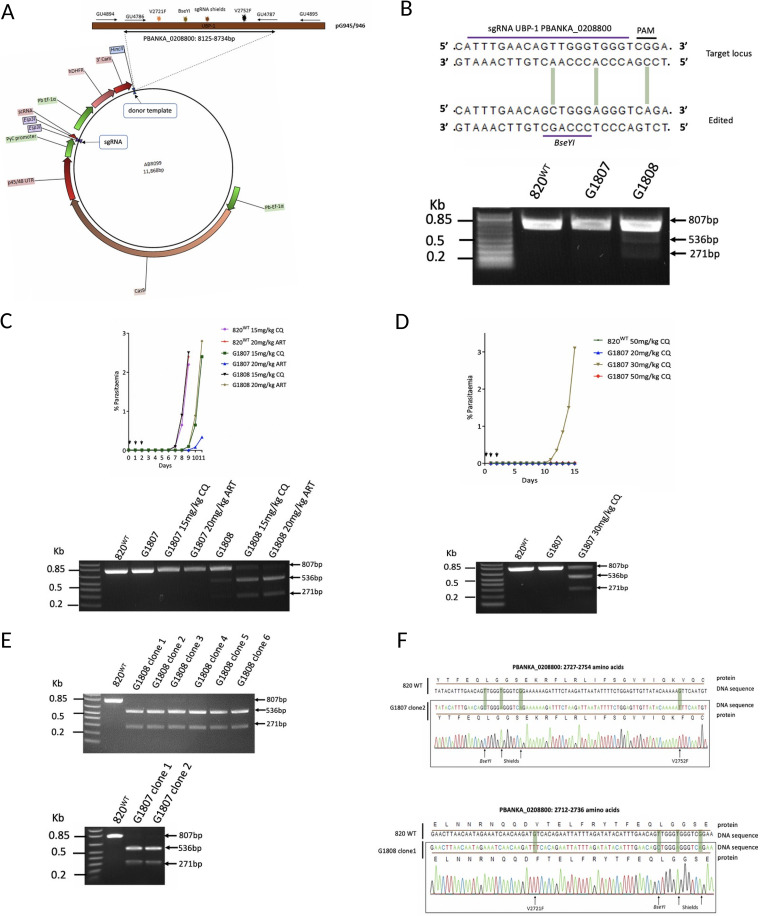
Introduction of UBP-1 mutations in P. berghei. (A) Schematic plasmid constructs for the UBP-1-targeted gene editing to introduce the V2721F and V2752F mutation. The plasmid contains Cas9 and *hdhfr* (for pyrimethamine drug selection) under the control of the P. berghei EF-1α promoter and the sgRNA expression cassettes under the control of the PyU6 promoter. A 20-bp guide RNA was designed and cloned into the sgRNA section of the illustrated vector. The donor UBP-1 sequence (610 bp) is identical to that of the wild type, albeit with the desired mutations of interest (indicated by colored star symbols): V2752F (pG945), V2721F and V2752F (pG946), and silent mutations that mutate the Cas9 binding site as well as introduce the restriction site BseYI for restriction fragment length polymorphism (RFLP) analysis. (B) Illustrated 20-bp sgRNA and RFLP analysis of mutant parasites. Successful editing in the transfected parasites was observed on day 12 after transfection and pyrimethamine drug selection. RFLP (BseYI digestion) analysis of the transformed line PCR products (primers GU4894 + GU4895, 807 bp) revealed ∼0.5% and ∼22% efficiency for the G1807 and G1808 lines, respectively, as indicated by 2 distinct bands (536 bp and 271 bp) compared to 807-bp bands in the parent 820 line. (C) Preemptive challenge of the G1807 and G1808 lines with ART and CQ at 20 mg/kg and 15 mg/kg, respectively, and RFLP analysis of recrudescent parasites. Mice were infected intraperitoneally (i.p.) with ∼2 × 10^7^ parasites on day 0. Treatment was started ∼4 h postinfection by i.p. injection for three consecutive days. Parasitemia was monitored by microscopy analysis until recrudescence was observed. (D) Preemptive challenge of the G1807 line with higher doses of CQ and RFLP (BseYI digestion) analysis of the G1807 recrudescent population after challenge with 30 mg/kg CQ. (E) RFLP analysis of the cloned G1808 and G1807 ART- and CQ-challenged recrudescent parasites. (F) DNA sequencing confirming successful nucleotide editing for the G1807 clone 2 and G1808 clone 1 lines. The top sequence represents the wild-type 820 line (820^WT^) unedited sequence with positions for sgRNA, protospacer adjacent motif (PAM), and V2721F or V2752F mutations indicated. The bottom sequence illustrates the nucleotide replacements at the V2721F or V2752F mutation locus and silent mutations to prevent Cas9 retargeting, as well as to introduce the BseYI restriction site for RFLP analysis in the G1807^V2752F^ and G1808^V2721F^ lines.

To experimentally demonstrate that UBP-1 mutations confer selective advantage upon ART pressure, we introduced P. chabaudi UBP-1 candidate mutation (V2697F and V2728F) equivalents (see Fig. S1 in the supplemental material) into the P. berghei 820 line using a CRISPR-Cas9 system developed and optimized in our lab (Fig. 1A). Two plasmids were initially designed to either introduce the single mutation, V2752F (V2728F P. chabaudi equivalent), or both mutations, V2721F (V2697F P. chabaudi equivalent) and V2752F, in an attempt to generate a double mutant (Fig. 1A). Silent mutations to mutate the Cas9 cleavage site and introduce a restriction site (BseYI) were also introduced to prevent retargeting of mutated loci by Cas9 for the former and diagnosis by restriction fragment length polymorphism (RFLP) for the latter (Fig. 1A and B). Transfections of these plasmids into the 820 line yielded ∼0.5% mutants for the V2752F mutant line (G1807, pG945) and ∼23.00% mutants for the V2721F and V2752F double-mutant line (G1808, pG946), as confirmed by RFLP analysis (BseYI digestion) of the edited UBP-1 locus (Fig. 1B). Since the efficiency was too low to clone out the mutant lines by serial dilution, we attempted a preemptive drug selection with CQ and ART of the G1807 and G1808 lines to examine if selective enrichment of the mutant population could be achieved. Indeed, after infecting mice with the G1808 line and treating for three consecutive days with ART at 20 mg/kg, the recrudescent parasite population on day 9 was enriched to ∼90% mutant population, as confirmed by RFLP analysis (Fig. 1C; see also Fig. S2D in the supplemental material). Meanwhile, CQ at 15 mg/kg also enriched the G1808 line to ∼80%, relatively less than did ART (Fig. 1C). On the contrary, a very low-level mutant enrichment of the G1807 line (0.5% to 2.6%) was observed with CQ at 15 mg/kg, while ART did not produce any enrichment in the same line (0.5%). Interestingly, cloning of the G1808 ART-enriched lines yielded six clones that were all single mutants positive for the V2721F mutation despite coming from a plasmid with donor templates that carried both the V2721F and V2752F mutations (Fig. 1E and F). This suggests that the single V2721F mutation-carrying parasites were predominant in the G1808 line (despite resulting from transfection with a plasmid carrying both mutations) and were selectively enriched by ART. These data also suggested that introducing both mutations into the same parasite could either be lethal or result in very unfit parasites that are easily cleared by the host during early growth following transformation. Indeed, bulk DNA sequence analysis of the G1808 uncloned line revealed the absence of traces for both mutations, as only parasites carrying V2721F with silent mutations were present (Fig. S2B). Sequence analysis of the G1808 line isolated after CQ challenge at 15 mg/kg also confirmed specific enrichment for the V2721F mutation (Fig. S2D), suggesting that despite being principally enriched by ART, the V2721F mutation also modulates some resistance to CQ. Meanwhile, when we challenged the G1807 line (V2752F single mutation) with CQ at higher doses (20, 30, and 50 mg/kg), a recrudescent population was observed on day 10 with CQ 30 mg/kg (Fig. 1D). The CQ 30 mg/kg recrudescent parasites were enriched to ∼61% for the mutant population (Fig. 1D, Fig. S2C) and were subsequently cloned. Sanger sequencing of G1808 ART-enriched and G1807 CQ-enriched clones confirmed the presence of the single V2721F and V2752F mutations, respectively, as well as the Cas9 cleavage silencing mutations and the silent mutations introducing the BseYI diagnostic restriction site (Fig. 1F).

### The V2721F mutation confers observable reduced *in vivo* susceptibility to ARTs, while the V2752F mutation confers resistance to CQ and low-level protection against ARTs.

**FIG 2:**
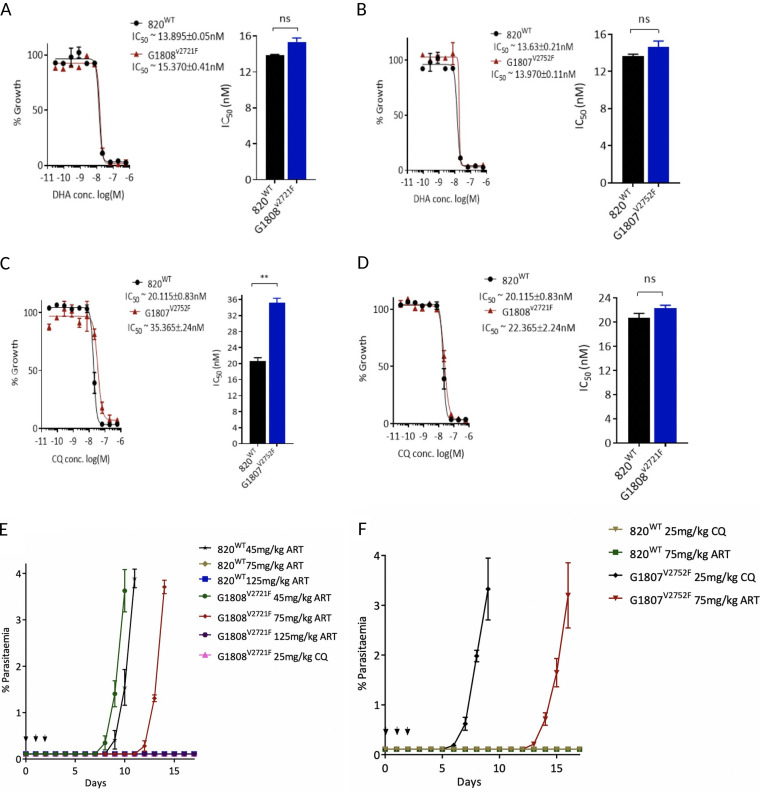
ART and CQ *in vitro* and *in vivo* resistance profiles of the G1807^V2752F^ and G1808^V2721F^ lines. dihydroartemisinin (DHA) dose-response curves and half-inhibitory concentration (IC_50_) comparisons of the G1808^V2721F^ (A) and G1807^V2752F^ (B) lines relative to that of the wild-type 820 line. CQ dose-response curves and IC_50_ comparisons of the G1807^V2752F^ (C) and G1808^V2721F^ (D) lines relative to that of the wild-type 820 line. Significant differences between mean IC_50_ values or IC_50_ shifts were calculated using the paired *t* test. Error bars are standard deviations from three biological repeats. Significance is indicated with asterisks as follows: *, *P* < 0.05; **, *P* < 0.01; ***, *P* < 0.001; ****, *P* < 0.0001; ns, not significant. Modified Peters’ 4-day suppressive test to monitor resistance to ART and CQ *in vivo* in the G1808^V2721F^ (E) and the G1807^V2752F^ (F) mutant lines. Groups of three mice were infected with 1 × 10^6^ parasites on day 0. Treatment started ∼1.5 h later with indicated drug doses every 24 h for three consecutive days (treatment days shown by arrows). Parasitemia was monitored by microscopy analysis of Giemsa-stained blood smears up to day 18. Error bars are standard deviations of parasitemia values from 3 mice.

**FIG 3:**
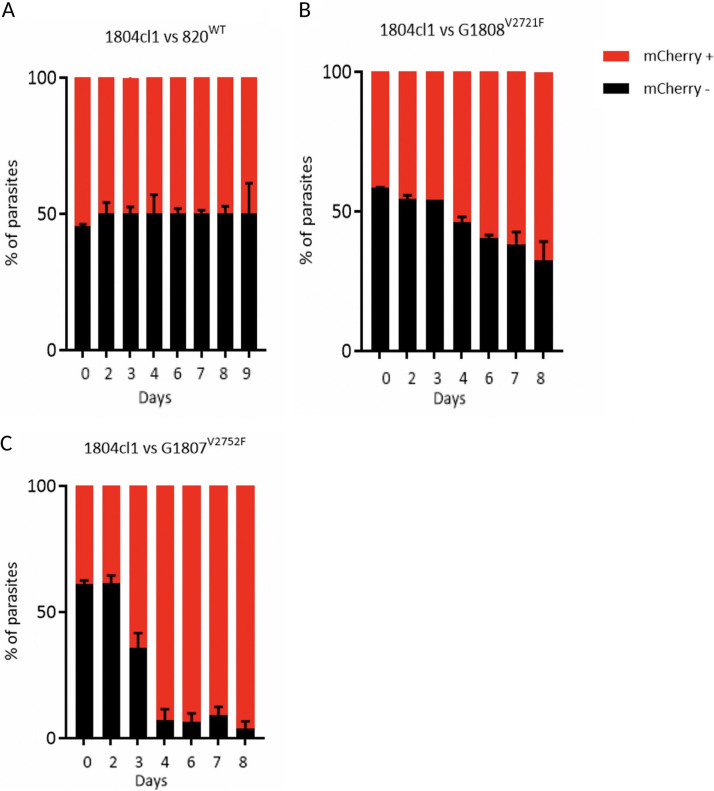
Growth kinetics of the 820, G1808^V2721F^, and G1808^V2752F^ lines relative to the 1804cl1 line. The 1804cl1 line constitutively expresses mCherry under the control of the *hsp70* promoter. The 820, G1808^V2721F^, and G1808^V2752F^ lines were mixed with the 1804cl1 line at a 1:1 ratio and injected intravenously at a parasitemia of 0.01% on day 0. Daily percentages of representative parasitemia of the 820 or mutant lines in the competition mixture were quantified by subtracting the total parasitemia based on positivity for Hoechst DNA stain from the fraction of the population that is mCherry positive (1804cl1) as determined by flow cytometry. On day 4, when parasitemia was ∼5%, blood from each mouse was passaged into a new naive host, and parasitemia was monitored until day 8. Percent population changes of the mutant and wild-type lines relative to the 1804cl1 line in the 820 (A), G1808^V2721F^ (B), and G1807^V2752F^ (C) lines. Error bars are standard deviations from three biological repeats.

We next quantitated the drug response profiles of the G1808^V2721F^ and G1807^V2752F^ cloned lines (first clone in each of the lines) *in vitro* and *in vivo* using DHA, ART, and CQ. In short-term P. berghei *in vitro* drug assays, both the G1808^V2721F^ and G1807^V2752F^ parasites showed no difference in sensitivity to DHA compared to that of the parental 820 line (Fig. 2A and B). The lack of decreased drug sensitivity of both lines is consistent with the failure of the standard 72-h drug assays to differentiate similar Kelch-13 ART-resistant parasites from sensitive lines in P. falciparum (3, 6). Meanwhile, a 1.8-fold increase in the half-inhibitory concentration (IC_50_) was observed for the G1807^V2752F^ line when challenged with CQ (Fig. 2C), but not for the G1808^V2721F^ line (Fig. 2D). However, rodent malaria parasites offer the advantage of experimental drug resistance assessment *in vivo*. Therefore, we profiled the *in vivo* drug responses of the mutant lines to parental ART, which with controlled parasite inocula has been shown to effectively suppress wild-type parasites for up to 18 days following 100 mg/kg dosing for three consecutive days (12). This is unlike responses to the clinically relevant ART derivative artesunate, which permits recrudescence in wild-type rodent malaria parasites at doses as high as 300 mg/kg within 14 days (20). This approach, when applied to the G1808^V2721F^ line, demonstrated that this mutation does indeed confer enhanced *in vivo* tolerance to ARTs compared to that of the parental 820 line. G1808^V2721F^ parasites survive three consecutive doses of 75 mg/kg ART, with the recrudescent population appearing on day 9 after the last dosing, whereas 820 wild-type parasites are effectively suppressed up to day 17 of follow-up (Fig. 2E). Both the G1808^V2721F^ and 820 lines survived a 45 mg/kg dose of ART, with the former having a slightly faster recrudescence rate on day 7, while the latter recrudesced a day later (Fig. 2E). Even though ART at 45 mg/kg does not significantly separate wild-type from mutant parasites, this could be due to the fitness cost that the V2721F mutation carries (Fig. 3), which would explain the recrudescence of mutant parasites at almost the same time as that for the wild type, since they would require a slightly longer time to achieve quantifiable parasitemia. Both lines remain sensitive to a 125 mg/kg ART dose, with no recrudescence observed up to day 17 (Fig. 2E). In contrast, the G1807^V2752F^ line is relatively resistant to CQ *in vivo* (Fig. 2F), surviving three consecutive doses at 25 mg/kg, with recrudescent parasites coming up on day 4 after the last dose, unlike the parental 820 line and the G1808^V2721F^ lines, which are sensitive and are effectively suppressed up to day 17. Interestingly, the G1807^V2752F^ line also displays low-level reduced susceptibility to ART at 75 mg/kg dose, with parasites coming up on day 12, later than in the G1808^V2721F^ line (Fig. 2F). These data confirm that the V2721F mutation confers protection from ART drug challenge, while the V2752F mutation mediates resistance, primarily to CQ and, to some extent, low-level protection to ARTs. The recrudescence of the wild-type 820 and G1808^V2721F^ parasites at 45 mg/kg ART is also in agreement with our previous finding that P. berghei is less sensitive to ARTs, especially in the spleen and bone marrow, which could be the source of recrudescent infection at relatively lower doses (21).

### Growth of parasites carrying UPB-1 V2752F and V2721F mutations is impaired.

The spread of drug resistance, as is the case in most microbial pathogens, is partly limited by detrimental fitness costs that accompany acquisition of such mutations in respective drug transporters, enzymes, or essential cellular components. The G1807 and G1808 lines carrying UBP-1 V2721F and V2752F mutations, respectively, were each grown in competition with a parental line expressing mCherry *in vivo* and were shown to be characteristically slow growing (Fig. 3A to C). In comparison, the G1807^V2752F^ line is severely impaired relative to the G1808^V2721F^ line, being completely outcompeted by day 8. These data and the earlier failure to generate the double mutant (Fig. 1) demonstrate that UBP-1 is an important (possibly essential) protein for parasite growth and that acquisition of resistance through mutation of UBP-1 confers mutation-specific fitness costs.

### Reversal of the V2752F mutation restores CQ sensitivity in the G1807^V2752F^ line, while introduction of the V2721F in the same line appears to be lethal.

**FIG 4:**
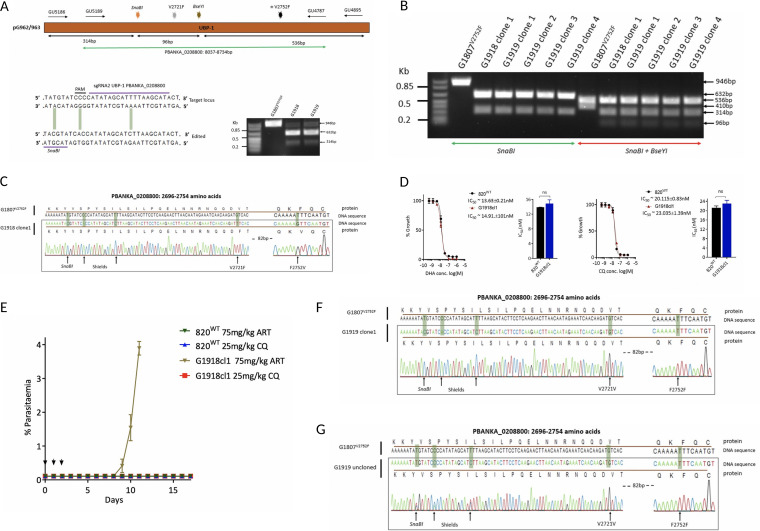
Swapping of the V2752F to V2721F mutations and attempted generation of a double mutant in the G1807^V2752F^ line. (A) Schematic of the UBP-1 donor DNA in the pG962 and pG963 vectors, a 20-bp guide RNA used to target the UBP-1 region upstream of the V2721F mutation in the Cas9-expressing vectors, with introduced silent mutation sites indicated. RFLP (SnaBI digestion) analysis of PCR products (GU5186 + GU4895, 946 bp) of the G1918 and G1919 lines relative to the mutants showing successful editing by 2 distinct RFLP bands for the mutants (632 bp and 314 bp) and residual traces of the parental genotype. (B) RFLP analysis of the cloned G1918 and G1919 lines. The first six lanes to the left are RFLP analyses of G1918 and G1919 cloned line PCR products (GU5186 + GU4895, 946 bp) digested by SnaBI, showing 2 bands (632 bp and 314 bp) compared to 1 band for the parental G1807^V2752F^ line. Six lanes to the right are the same clones digested by both SnaBI and BseYI, showing parental G1807^V2752F^ with 2 RFLP bands (536 bp and 410 bp) as a result of digestion with BseYI only, as the SnaBI restriction site is absent, and 3 RFLP bands (536 bp, 314 bp, and 96 bp) in the G1918 and G1919 clones as a result of digestion of the PCR product by both BseYI and SnaBI. (C) Sequencing of G1918 clone 1 showing successful swapping of V2752F in the parent G1807^V2752F^ line to the V2721F mutation. (D) *In vitro* DHA and CQ dose response curves and IC_50_ comparisons of the G1918cl1 revertant line relative to those of the wild type, showing reversion of the CQ phenotype and similar sensitivity to DHA. Significant differences between mean IC_50_ values or IC_50_ shifts were calculated using a paired *t* test. Error bars are standard deviations from three biological repeats. Significance is indicated with asterisks as follows: *, *P* < 0.05; **, *P* < 0.01; ***, *P* < 0.001; ****, *P* < 0.0001; ns, not significant. (E) *in vivo* tolerance to ARTs at 75 mg/kg in the G1918cl1 line and complete restoration of CQ sensitivity. Sequence analysis of the G1919 clone1 (F) line and the G1919 uncloned (G) line, showing absence of double mutant populations.

Drug pressure can select, in the long or short term, for mutations in sensitive parasite populations that would affect responses to the same drug. To further confirm that the phenotypes observed in our mutant lines were due to the V2721F or V2752F mutations and not to possible secondary mutations that may have been acquired during the preemptive drug pressure, we attempted to reverse the V2752F mutation in the G1807^V2752F^ line by swapping it to the V2721F genotype. This would allow us to determine if wild-type CQ phenotypes can be restored in the G1807^V2752F^ line, while at the same time assessing if the ART susceptibility profiles of the G1808^V2721F^ mutants could be reproduced in an independent line. Using a CRISPR-Cas9 editing strategy similar to the one outlined above, a single guide RNA (sgRNA) targeting a region ∼50 bp upstream of the V2721F mutation was designed and cloned in the Cas9-expressing vectors (Fig. 4A). Donor DNA (698 bp; GU5189 + GU4787) containing the V2721F (for targeted mutation swap) or both the V2721F and V2752F mutations (for a forced introduction of V2721F in the G1807^V2752F^ background) was used to generate the vectors pG963 and pG962, respectively (Fig. 4A). Silent mutations mutating the protospacer adjacent motif (PAM) site, as well as introducing a second restriction site, SnaBI, for RFLP analysis were also included. Transfection of the G1807^V2752F^ line with pG963 and pG962 vectors successfully edited the UBP-1 locus, generating the G1918 and G1919 lines, respectively, with ∼88% and ∼79% efficiency as confirmed by SnaBI RFLP analysis (Fig. 4A). Cloning and sequencing of the G1918 line revealed a successful targeted mutation swap, introducing the V2721F mutation and reediting the 2752F to 2752V wild-type genotype (Fig. 4B and C). Phenotype analysis of the G1918 clone line revealed a restored *in vitro* susceptibility to CQ similar to that of the 820 wild type and a similar DHA sensitivity (Fig. 4D). Under *in vivo* conditions, the G1918cl1 line displayed a similar ART susceptibility profile at 75 mg/kg as the G1808^V2721F^ line, while CQ sensitivity was completely restored (Fig. 4E). This provided further experimental evidence that the drug susceptibility profiles observed were due to the V2721F or V2752F amino acid substitutions and not to the introduced silent mutations or secondary mutations that may have been acquired during the preemptive drug exposure. Interestingly, cloning and sequencing of the G1919 (Fig. 4B and F) line revealed successful introduction of the silent mutations (PAM mutating and SnaBI); the V2721F mutation was absent in all four clonal lines, yet the parental V2752F mutation was retained. This suggested that introduction of V2721F in the V2752F background is lethal or refractory in the parasite and further supported our failed first attempt to generate the double-mutant line (Fig. 1). Detailed sequence analysis of the transfected parasite populations before cloning revealed the presence of only one mutation trace in the G1919 line (despite the donor DNA containing both mutations), confirming that the double-mutant parasites do not survive or are severely growth impaired and quickly overgrown by the single-mutation parasites (Fig. 4G).

## DISCUSSION

Ubiquitin hydrolases or deubiquitinating enzymes (DUBs) are essential elements of the eukaryotic ubiquitin proteasome system (UPS), which is primarily involved in maintaining cellular protein homeostasis and responding to stress. Despite the proposed involvement of Plasmodium DUBs in modulating susceptibility to multiple drugs, lack of conclusive experimental evidence has thus far limited studies into their detailed involvement in mode of action and or resistance phenotypes, such as those observed with ARTs. In this study, using a CRISPR–Cas9-mediated reverse genetics approach, we have provided experimental evidence on the direct involvement of a DUB (UBP-1) in modulating parasite responses to ART and CQ, most importantly under *in vivo* conditions. As the debate into the mechanism of action and resistance to ARTs continues, a consensus understanding is converging that ART resistance is complex, as several factors, genetic determinants, and possibly mechanisms of action appear to be involved. In P. falciparum, ART resistance is confined to early ring stage parasites, which has been translated in laboratory conditions to increased survival in ring stage survival assays (RSAs) (6). Mutations in Pfkelch13 and PfCoronin, as well as transient (hypothermic-hyperthermic) temperatures, have been shown to enhance ring stage parasite survival in the RSAs (10, 22, 23). More recently, characterization of Kelch-13 interacting factors has revealed that disruption of proteins that colocalize with Kelch-13, such as the parasite endocytosis proteins ESP15, UBP-1, and others of unknown function, modulate susceptibility to ARTs (24). As demonstrated in this study, reduced ART and, more, reduced CQ susceptibility can be mediated by mutations in UBP-1, underscoring a potential mechanism of cross resistance and some commonality in mode of action between CQ and ART, especially relating to hemoglobin digestion and trafficking in malaria parasites (24–26).

The UBP-1 V2728F mutation was previously designated a principle determinant of reduced ART susceptibility despite its common fixation with mefloquine and higher doses of CQ (12). Contrary to this argument, ART did not enrich this mutation (V2752F) in our study, but instead enriched the V2721F mutation, which was fixed with artesunate in P. chabaudi. However, enrichment of the V2752F mutation with a higher dose of CQ was achieved, showing that this mutation does indeed modulate parasite responses to CQ, while the V2721F mutation is chiefly responsible for the reduced ART susceptibility phenotype in the P. berghei model *in vivo*. Interestingly, drug challenge of these mutant lines *in vivo* revealed that both mutations give low-level cross-protection against both ARTs and CQ. This confirms that each of these UBP-1 mutations modulates some form of protection to both ARTs and CQ drug challenges, albeit to differing degrees, which is, therefore, in strong agreement with previous observations in P. chabaudi (12). This also demonstrates a plurality of pathways to resistance involving the same target. Recently, the exact equivalent UBP-1 mutations in P. falciparum, V3275F and V3306F, have been successfully engineered (15). In P. falciparum UBP-1, the V3275F mutation (V2721F P. berghei equivalent) shows enhanced survival to DHA in RSAs but remains sensitive to CQ. However, unlike in P. berghei, the V3306F (V2752F P. berghei equivalent) showed no enhanced survival to DHA in RSAs or resistance to CQ (15). While not entirely in agreement with the data reported here, this could be due to limitations in the ability of *in vitro* assays to fully predict actual drug responses *in vivo*, which our data highlight and which has been a concern recently with Kelch-13 mutations (27). These observations may also somewhat be confounded by species-specific differences in drug responses, pharmacodynamics, modes of action, and resistance that, in part, remain to be fully investigated. For example, previous and original linkage studies in P. chabaudi identified additional mutations in an amino acid transporter (*pcaat*), in tandem with UBP-1 mutations, as being strongly associated with CQ resistance phenotypes (12). Even though this could partly explain the observed *in vitro* sensitivity of P. falciparum V3275F mutants to CQ, our data suggest that UBP-1 mutations are sufficient to mediate quantifiable protective phenotypes to both ARTs and CQ, as the reversal of the V2752F mutation performed in this study, for example, completely restores CQ sensitivity. This provides, therefore, additional independent evidence on the direct causative role of UBP-1 mutations in modulating parasite responses not just to ARTs, but to CQ as well. The study also illustrates the potential of the P. berghei rodent model in proving causality to antimalarial drug resistance phenotypes under *in vivo* conditions, especially in light of recent reported discrepancies between some *in vitro* RSA resistance profiles of P. falciparum Kelch-13 mutants and actual *in vivo* phenotypes using the *Aotus* monkey model (27).

Interestingly, the V2721F and V2752F mutation-carrying parasites are characteristically slow growing and are easily outcompeted in the presence of nonmutants. Natural P. falciparum UBP-1 mutations have been reportedly associated with ART treatment failure in Kenya (16, 19), SEA (18), and, more recently, in Ghana (17) (see Fig. S4 in the supplemental material). However, unlike their rodent counterparts, which are associated with reduced ART susceptibility, the reported natural E1528D and D1525E mutations occur toward the less conserved N terminus of the protein and outside the conserved, bioinformatically predicted UBP-1 catalytic domain (11) (Fig. S1). This suggests that acquisition of the mutations at the well-conserved C terminus in P. falciparum leads to a potential growth defect, as we observed with P. berghei in this study. However, as these upstream mutations are not conserved between P. falciparum and P. berghei UBP-1, we cannot test the hypothesis in this model. In fact, P. falciparum UBP-1 is highly polymorphic, with over 480 reported single-nucleotide polymorphisms (SNPs) (https://plasmodb.org), all of which are in the N-terminal region. *P. falciparum* UBP-1 has also been recently shown to be undergoing a strong positive selection in SEA (28). UBP-1 mutations could, therefore, be an independent avenue by which ART or multidrug resistance phenotypes could emerge in regions where malaria is endemic, as has been seen in Africa (Ghana and Kenya), without actually requiring a permissive genetic background, as seems to be the current landscape with Kelch-13 mutations. However, there are constraints upon the evolution of drug resistance and UBP-1. While these data confirm that a single protein that does not transport drugs can mediate resistance to two quite distinct drug entities, it was not possible to generate a P. berghei line that simultaneously contained the two UBP-1 drug resistance mutations examined in this study.

In yeasts, UBP-1 localizes to the endoplasmic reticulum and plays a role in protein transport, specifically in internalization of substrates across membranes (29). Mutations in UBP-1 could, therefore, modulate endocytosis of important essential host-derived products such as hemoglobin to the digestive vacuole in a similar manner, thereby reducing exposure of the parasite to activated drug for both ARTs and CQ. Interestingly, mutations in the AP2 adaptor complex that is involved in clathrin-mediated endocytosis have also been implicated in ART resistance in rodent malaria parasites (14). One of the AP2 adaptor complex mutations (I592T) has been recently engineered in P. falciparum and has been shown to enhance ring stage parasite survival in RSAs (15). This further suggests that inhibition of the endocytic trafficking system is a possible generic mechanism for the parasites to survive lethal doses of drugs that require transport and activation in the digestive vacuole. This would further explain the multidrug resistance phenotype observed with the UBP-1 mutations in P. chabaudi and P. berghei in this study. Acquisition of the V2728F mutation in P. chabaudi was structurally predicted to reduce deubiquitination (11). In such a situation, the cellular increase in ubiquitinated proteins would be anticipated to positively feedback to the cellular machinery to rapidly degrade protein substrates at the 20s proteasome, promoting nonspecific and rapid protein turnover or impaired substrate trafficking. This would result in generally slow-growing parasites with reduced expression of, for example, multidrug resistance transporters, as well as reduced endocytosis of host-derived products like hemoglobin, which would in turn modulate parasite responses to these drugs. More recently, functional studies have revealed that PfKelch13 (a known determinant of ART resistance) localizes to the parasite cytostome and plays a role in hemoglobin trafficking (24, 26). Consequently, PfKelch13 mutations have been shown to lead to a partial loss of PfKelch13 protein function, leading to decreased hemoglobin trafficking to the parasite digestive vacuole and less DHA activation, which in turn mediates parasite survival (24, 26). Strikingly, protein pulldown at the parasite cytostomal foci where Kelch-13 localizes identified UBP-1 as a key interacting partner in the Kelch-13-mediated endocytic machinery that is involved in hemoglobin trafficking. By analyzing hemoglobin endocytosis in the ring and trophozoite stages, it has been demonstrated that partial inactivation of UBP-1 impairs hemoglobin endocytosis in both rings and trophozoites, unlike inactivation of Kelch-13, which impairs hemoglobin uptake only in ring stages of the parasites (24). This is indeed in agreement with our hypothesis on the consequences of UBP-1 mutations, and with observed P. berghei phenotypes, which in a similar manner could impair trafficking of hemoglobin, leading to less activation of ARTs and CQ. Moreover, the potential role of UBP-1 in trafficking hemoglobin in both rings and trophozoites could explain the ART and CQ potential cross-resistance phenotype that we have observed with UBP-1 mutations unlike with Kelch-13 mutations, which, thus far, are known to mediate resistance to ARTs only and only in early ring stages. The experimental validation on the involvement of UBP-1 mutations in mediating potential cross-resistance to ART and CQ in malaria parasites, therefore, provides an additional understanding of drug resistance in malaria parasites, specifically for compounds that require access and/or activation in the digestive vacuole. Furthermore, the P. berghei model provides a useful sensitive and robust system in which to investigate the interplay and impact of simultaneous mutations of both Kelch-13 and UBP-1 *in vivo*, as well as to assess whether PfKelch13 mutations would modulate responses to CQ under *in vivo* conditions.

In conclusion, the work presented here provides further experimental evidence for the involvement of conserved mutations in a polymorphic ubiquitin hydrolase protein that serves as a nexus for resistance to two very diverse classes of drugs. The findings also underscore the potential difficulties that *in vitro* assays may have in appropriately assigning mutant parasites with appropriate phenotypes in the absence of conclusive *in vivo* measurements. P. berghei should therefore, be a suitable and adaptable *in vivo* model for the rapid evaluation and/or genetic engineering of mutations associated with human-infectious *Plasmodium* drug resistance observed in the field for concurrent assigning of drug resistance phenotypes under both *in vitro* and *in vivo* conditions.

## MATERIALS AND METHODS

### CRISPR-Cas9 generation of UBP-1 mutant lines.

**(i) Primary vectors.** The Cas9-expressing plasmid ABR099 was used for targeted nucleotide replacement at the UBP-1 locus. ABR099 (Fig. 1A) contains the Cas9 endonuclease driven by the P. berghei Ef-1α promoter, a Cas9 binding scaffold, a site for cloning the guide RNA (sgRNA) driven by the Plasmodium yoelii U6 promoter, an *hdhfr* cassette (for pyrimethamine drug resistance selection), and a linker site for insertion of homologous repair templates. sgRNAs targeting the UBP-1 locus were designed using the Web-based eukaryotic pathogen CRISPR guide RNA/DNA design tool (http://grna.ctegd.uga.edu/) (30) by directly inputting the sequence of interest. Primary vectors containing the sgRNA of interest were generated by annealing the oligonucleotide pairs (GU4788+GU4789 and GU5206+GU5207; see Table S1 in the supplemental material) encoding the guide sequence and cloning them into the dual Esp3I sites upstream of the Cas9 binding domain of the vector ABR099. These plasmids were called pG944 and pG960 for the GU4788 + GU4789 and GU5206 + GU5207 annealed guides, respectively.

**(ii) Mutagenesis and generation of secondary vectors.** To generate the final vectors for editing the UBP-1 locus, 610 bp of UBP-1 donor DNA (PlasmoDB gene ID PBANKA_0208800) was PCR amplified using primers GU4786 and GU4787 (Table S1) designed to contain a HincII site at the 5′ end. The PCR product was purified, A tailed, and cloned into the TOPO 2.1 vector using the TOPO TA cloning kit (Invitrogen) according to the manufacturer’s instructions. To mutate the UBP-1 locus, 3 primer sets (Table S1) complementary to the amplified UBP-1 PCR product were designed to contain specific nucleotide substitutions, as follows: (i) a shielding primer (GU4783) containing three silent mutations mutating the sgRNA and PAM sites targeted by the GU4788 + GU4789 sgRNA (to prevent Cas9 binding the donor templates and the edited loci in the mutant parasites), as well as an introduced BseYI restriction site for restriction site fragment polymorphism (RFLP) analysis, and (ii) 2 primer sets carrying the mutations of interest, V2721F (GU4785) and V2752F (GU4784). A site-directed mutagenesis of the cloned UBP-1 PCR product in the TOPO 2.1 vector was carried out using a QuikChange multisite-directed mutagenesis kit (Agilent Technologies) using the following primer combinations: GU4783 + GU4784 for the V2752F single mutant and GU4783 + GU4784 + GU4785 for the double mutant. The resulting mutant fragments in the TOPO 2.1 vector were digested out and cloned into the linker site of the vector pG944 using the HincII restriction site to generate pG945 (single mutant) and pG946 (double mutant). For targeted mutation swapping and a second attempt to generate a double mutant line, a second sgRNA (GU5206 + GU5207) upstream of the V2721F mutation was designed and cloned into the ABR099 vector as described. Donor DNA was amplified from the G1808^V2721F^ or pG946 vector to generate single- or double-mutation templates, respectively, by using overlapping PCR as previously described (31). Briefly, internal complementary primers (GU5190 + GU5191; Table S1) carrying 3 silent mutations (2 for mutating the sgRNA and PAM of the GU5206 + GU5207 sgRNA and 1 to introduce the SnaBI restriction site for RFLP analysis) were used to amplify 2 overlapping PCR products from the G1808^V2721F^ DNA or pG946 plasmid upon linkage to HincII, introducing outer primers GU5189 and GU4787 (Table S1). After gel purification, ∼50 ng of the overlapping PCR fragments was used as the template in a second round of PCR using the two outer primers (GU5189 + GU4787) to generate donor fragments with mutations of interest. The resulting fragments were subsequently cloned into the pG960 vector at the linker site using the HincII restriction site to generate the vectors pG963 (silent mutations to GU5206 + GU5207 sgRNA, V2721F mutation) and pG962 (silent mutations to GU5206 + GU5207 sgRNA, V2721F and V2752F mutation). All PCRs were carried out using the Kapa high-fidelity PCR kit (Roche). Plasmids were verified by Sanger DNA sequencing prior to further use.

### P. berghei animal infections.

P. berghei parasites were maintained in female Theiler’s Original (TO) mice (Envigo) weighing between 25 and 30 g. Parasite infections were established either by intraperitoneal (i.p.) injection of ∼200 μl of cryopreserved parasite stocks or by intravenous (i.v.) injection of purified schizonts. Monitoring of parasitemia in infected mice was done by examining methanol-fixed thin blood smears stained in Giemsa (Sigma) or by flow cytometry analysis of infected blood stained with Hoechst 33342 (Invitrogen). Blood from infected mice was collected by cardiac puncture under terminal anesthesia. All animal work was performed in compliance with UK home office licensing (project reference no. P6CA91811) and with ethical approval from the University of Glasgow Animal Welfare and Ethical Review Body.

### Parasite lines and transfections.

An 820 line that express green fluorescent protein (GFP) and red fluorescent protein (RFP) in male and female gametocytes, respectively (32), was used for initial transfection experiments, while the 1804cl1 line, which constitutively expresses mCherry throughout the life cycle (33), was used for growth competition assays as a control. Episomal plasmid DNA (∼10 μg) from the vectors described above was transfected by mixing with Nycodenz-purified schizonts and electroporated using the Amaxa Nucleofector device II program U-o33 as previously described (34). Parasites were then immediately i.v. injected into the mouse tail vein. Positive selection of transfected parasites was commenced 24 h later by inclusion of pyrimethamine (Sigma) in drinking water.

### Genotype analysis of mutant lines.

Blood was collected from parasite-infected mice by cardiac puncture under terminal anesthesia and lysed by resuspension in 1× E-lysis buffer (Thermo). Parasite genomic DNA was extracted using the Qiagen DNeasy blood and tissue kit according to manufacturer’s instructions. Genotype analysis of the transfected or cloned parasite lines was conducted initially by dual PCR-RFLP. PCR using exterior primers (GU4894 + GU4895 or GU5186 + GU4895) was used to amplify fragments from the DNA of the mutant lines, followed by restriction digests with either BseYI or SnaBI restriction enzymes to verify successful editing of the UBP-1 locus. Transfection efficiencies were estimated by relative densitometric quantification of RFLP fragments by ImageJ2 (35). Further confirmation of the mutations was carried out by Sanger DNA sequencing.

### P. berghei *in vitro* culture and drug susceptibility assays.

For *in vitro* maintenance of P. berghei, cultures were maintained for one developmental cycle using a standardized schizont culture medium containing RPMI 1640 with 25 mM hypoxanthine, 10 mM sodium bicarbonate, 20% fetal calf serum, 100 U/ml penicillin, and 100 μg/ml streptomycin. Culture flasks were gassed for 30 s with a special gas mix of 5% CO_2_, 5% O_2_, and 90% N_2_ and incubated for 22 to 24 h at 37°C with gentle shaking, conditions that allow for development of ring stage parasites to mature schizonts. Drug assays to determine *in vitro* growth inhibition during the intraerythrocytic stage were performed in these standard short-term cultures as previously described (36). Briefly, 1 ml of infected blood with a nonsynchronous parasitemia of 3 to 5% was collected from an infected mouse and cultured for 22 to 24 h in 120 ml of schizont culture media. Schizonts were enriched from the cultures by Nycodenz density flotation as previously described (34), followed by immediate injection into a tail vein of a naive mouse. Upon i.v. injection, schizonts immediately rupture, with the resulting merozoites invading new red blood cells within minutes to obtain synchronous *in vivo* infection containing >90% rings and a parasitemia of 1 to 2%. Blood was collected from the infected mice 2 h postinjection and mixed with serially diluted drugs in schizont culture medium in 96-well plates at a final hematocrit of 0.5% in a 200-μl well volume. Plates were gassed and incubated overnight at 37°C. After 22 to 24 h of incubation, schizont maturation was analyzed by flow cytometry after staining the infected cells with the DNA dye Hoechst-33258. Schizonts were gated and quantified based on fluorescence intensity on an FACSCelesta or an LSRFortessa (BD Biosciences, USA). To determine growth inhibitions and calculate half-inhibitory concentrations (IC_50_), quantified schizonts in no-drug controls were set to correspond to 100% with subsequent growth percentages in the presence of drugs, calculated accordingly. Dose-response curves were plotted in GraphPad Prism 7.

### *In vivo* drug assays.

A modified Peters’ 4-day suppressive test was employed to assess *in vivo* drug responses and/or resistance profiles in the wild-type and mutant lines, as previously described (37). Parasitemia was initiated by i.p. inoculation of between 10^6^ and 10^7^ parasites, followed by three daily consecutive drug doses initiated ∼4 h postinoculation. CQ was prepared at 50 mg/ml in 1× phosphate-buffered saline (PBS) and diluted to working stock in 1× PBS, while ART was prepared at 12.5 mg/ml in a 1:1 mixture of dimethyl sulfoxide (DMSO) and Tween 80 (Sigma), followed by a 10-fold dilution in sterile water to an injectable working solution. All drugs were delivered by i.p. injection and were prepared fresh immediately before injection. Parasitemia was monitored daily by flow cytometry and analysis of methanol-fixed Giemsa stained smears.

### *In vivo* growth competition assays.

Clonal mutant lines in the 820 background were mixed with the 1804cl1 line, which constitutively express mCherry under the control of the *hsp70* promoter, in a 1:1 mixture and injected intravenously into mice. Parasitemia in the competition mixtures was quantified by flow cytometry quantification of mCherry-positive parasites for the 1804cl1 proportional percentage and by subtracting the total parasitemia (Hoechst positive) from the mCherry-positive proportion for the 820 control and or mutant lines. Differentiation of the mCherry-positive population from the RFP in the 820 line was carried out by applying flow compensation gating strategies (see Fig. S3 in the supplemental material).

## Supplementary Material

Supplemental file 1
