# Supplementary material for: Experimentally Engineered Mutations in a Ubiquitin Hydrolase, UBP-1, Modulate In Vivo Susceptibility to Artemisinin and Chloroquine in Plasmodium berghei
Source: Antimicrob Agents Chemother. 2020 Jun 23;64(7):e02484-19. doi: 10.1128/AAC.02484-19 (PMC7318008; doi:10.1128/AAC.02484-19)
Supplement: Supplemental file 1 [file AAC.02484-19-s0001.pdf]

Supplementary materials for

Experimentally engineered mutations in a ubiquitin hydrolase, UBP-1, modulate *in vivo* susceptibility to artemisinin and chloroquine in *Plasmodium berghei*.

Nelson V. Simwela[1], Katie R. Hughes[1], A. Brett Roberts[1], Michael T. Rennie[1], Michael P. Barrett[1], Andrew P. Waters[1]*

[1]Institute of Infection, Immunity & Inflammation, Wellcome Centre for Integrative Parasitology, University of Glasgow

* Corresponding author: Andrew P. Waters, email: Andy.Waters@glasgow.ac.uk

Supplementary figures legends

Supplementary table 1: List of primers used

Supplementary Figure 1: Cartoon of *P. berghei* UBP-1 and sequence alignment of *P. falciparum*, *P. chabaudi* and *P. berghei* at the C-terminal. A. *P. berghei* UBP-1 showing the predicted catalytic domain and localisation of the engineered mutations and their *P. falciparum P. chabaudi* equivalents. Positions of *P. falciparum* UBP-1 D1525E and E1528D mutations which have been reported in the field but are not conserved in *P. berghei* and *P. chabaudi* are indicated. **B**. Sequence alignment of *P. falciparum*, *P. chabaudi* and *P. berghei* at the conserved C-terminal. Mutation sites are indicated for *P. falciparum* and *P. chabaudi* on top and *P. berghei* on the bottom. Conserved sites are indicated by the * symbol.

Supplementary Figure 2: DNA sequencing and trace analysis of G1807 and G1808 CQ and ART challenged lines.

Sequence analysis of the G1807 **(A)** and G1808 **(B)** uncloned lines. Enrichment of the V2752F (**C**) by CQ at 30mg/kg and V2721F **(D)** mutations by CQ and ART.

Supplementary Figure 3: Flow cytometry gating strategy for growth competition experiments

Representative flow cytometry gating strategies for growth competitions of wild type and mutant UBP-1 lines on Day 3**.** Acquired events were plotted on a forward (FS) and side (SS) scatter. Gate A was drawn to exclude debris. Events from gate A were plotted on a FS vs FH where gate B was drawn to exclude cell clumps and potential doublets. Events from gate B were then plotted on Hoescht vs FS and Total PT gate was drawn to quantify total parasitaemia. Events from gate B were also plotted on mCherry vs RFP where the mCherry positive population was distinguished from RFP positive female gametocytes by applying compensation spill-over filters that allow discrimination of the two colours as illustrated in the plots. Parasitaemia of mutant parasites was quantified by subtracting the mCherry positive population from the total parasitaemia as quantified by Hoescht staining of parasite DNA.

Supplementary Figure 4: Distribution of ART and CQ resistance mutations in Africa and South East Asia.

CQ resistance is believed to have originated in SEA and some parts of South America and eventually spread to Africa (1). Current distribution of Kelch 13 mutations in SEA (2-4) and reported UBP-1 polymorphisms (5-8) .

Supplementary table1

Primer ID	Sequence (5’-3’)
**GU4783**	TACATTTGAACAGCTGGGAGGGTCAGAAAAAAGATTTC
**GU4784**	TTCTGGAGTTGTTATACAAAAATTTCAATGTCAAAAATG
**GU4785**	TAACAATAGAAATCAACAAGATTTCACAGAATTATTTAG
**GU4786**	cgttaacGATAGCTACACAAACCTTCTTTC
**GU4787**	cgttaacCTCATTTGAGGTAAATGACCAG
**GU4788**	tattGATTTGAACAGTTGGGTGGGT
**GU4789**	aaacACCCACCCAACTGTTCAAATC
**GU4894**	CCAAAGTTCCTCTAACATAATATCTATC
**GU4895**	CTGATGATGCTGATACACCAC
**GU5186**	CCCCTGTTGGTTTAATAAATTTAG
**GU5189**	cgttaacCCAAAGTTCCTCTAACATAATATCTATC
**GU5190**	GAATAAAAAATACGTATCACCATATAGCATCTTAAGCATAC
**GU5191**	GTATGCTTAAGATGCTATATGGTGATACGTATTTTTTATTC
**GU5206**	tattGTATGCTTAAAATGCTATAT
**GU5207**	aaacATATAGCATTTTAAGCATAC

Supplementary figure 1

A

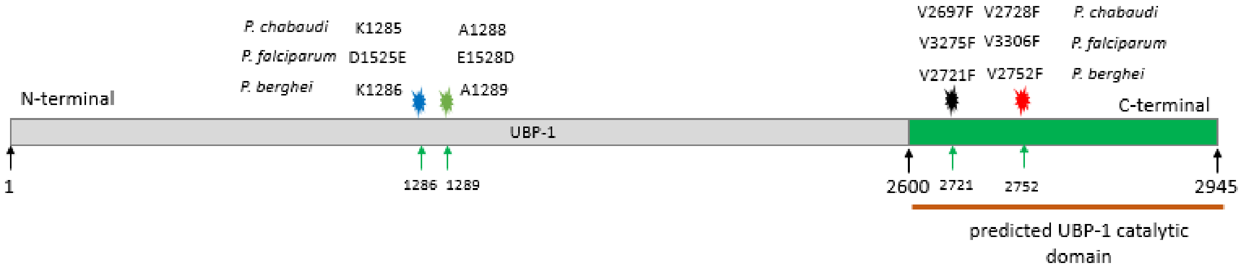

B

```
                                                                  V3275F, V2697F
PF3D7_0104300     NSELDYFLEEIKSFFKNMLTTDKSYISADRVLNMLPVELNNRNQQDVTEVFRYIFDKLGG     3288
PCHAS_0207200     NLLSKRFLYELKILFKLMTTTNKKYVSPDNILGILPQELNNRNQQDVTELFRYTFEQLGG     2710
PBANKA_0208800    NLLSKRFLYELKILFKLMSTTNKKYVSPYSILSILPQELNNRNQQDVTELFRYTFEQLGG     2734
                  *    . ** *:* :** * **:*.*:*    :*.:** ************.*** *::***
                                   V3306F, V2728F                  V2721F
PF3D7_0104300     SEKEFLRLIFSGVVIQKVQCQKCLFISKKKEIIHDLSFPVPISTNEKLSIQRFFDTFIQK     3348
PCHAS_0207200     SEKKFLRLIFSGVVIQKVQCQKCFFISKKEEIIHDLSFHVPAKSSKKQSIQKFFDTYIQK     2770
PBANKA_0208800    SEKRFLRLIFSGVVIQKVQCQKCFFISKKEEIIHDLSFHVPAKSTKKESIQKFFDTYIQK     2794
                  ***.*******************.******.******** **  .:.:* ***:****:***
                                   V2752F
PF3D7_0104300     EKIYGNNKYKCSRCNKKRNALKWNEIISPPCHLILILNRYNWSFSSNEKKKIKTHVKINS     3408
PCHAS_0207200     EKIYGNNKYKCSKCNKRRNALKWNEIISPPCHLILILNRYNWSFSSNEKKKIKTHVKINK     2830
PBANKA_0208800    EKIYGNNKYKCSKCNKRRNALKWNEIISPPCHLILILNRYNWSFTSNEKKKIKTHVKINK     2854
                  ************:***:**************************:**************.

PF3D7_0104300     KIVVNNFDYKLYGAIIHGGISASSGHYYFIGKKSERQNKKKSSWYQMNDSVVTKANSKMI     3468
PCHAS_0207200     KIVVNNFDYRLYGGIIHSGVSASSGHYYFIGKKSEKGDNSKNEWYQMDDSAITKVSSKSI     2890
PBANKA_0208800    KIVVNNFDYKLYGGIIHSGVSASSGHYYFIGKKSEKCDNSKNEWYQMDDSVITKVSSKSI     2914
                  *********:***.***.*:***************: ::.*..****:**.:**..** *

PF3D7_0104300     NKISKDLSNDHTPYVLFYRCKQAPISPDLYF     3499
PCHAS_0207200     NRISKDPSNDHTPYVLFYRCKQAPDSPSLYF     2921
PBANKA_0208800    NRISKDLSNDHTPYVLFYRCKQAPVSPSLYF     2945
                  *:**** ***************** **.***
```

Supplementary figure 2

A

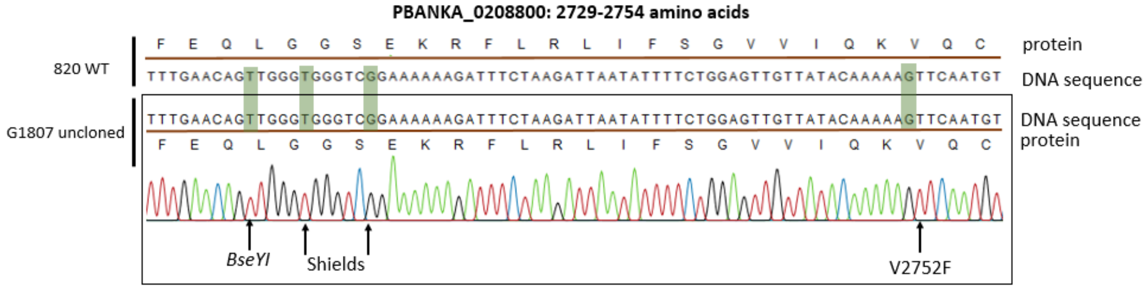

B

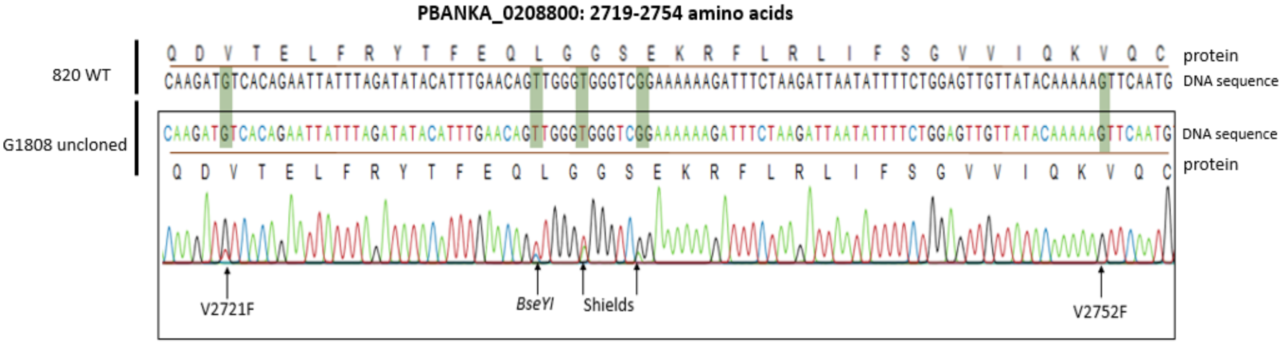

C

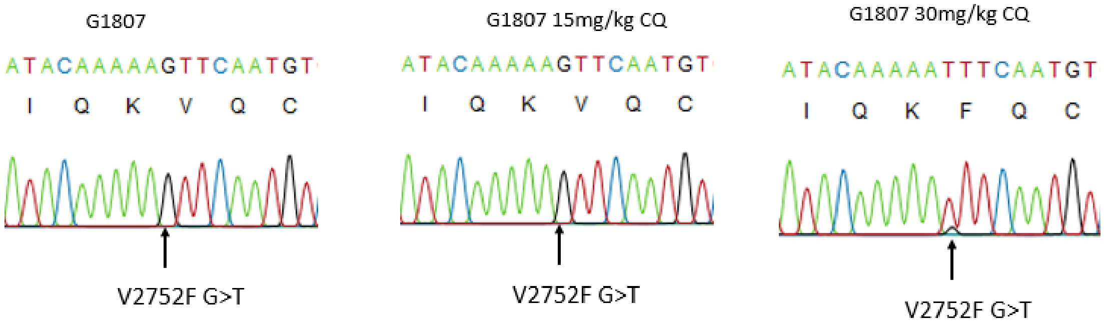

D

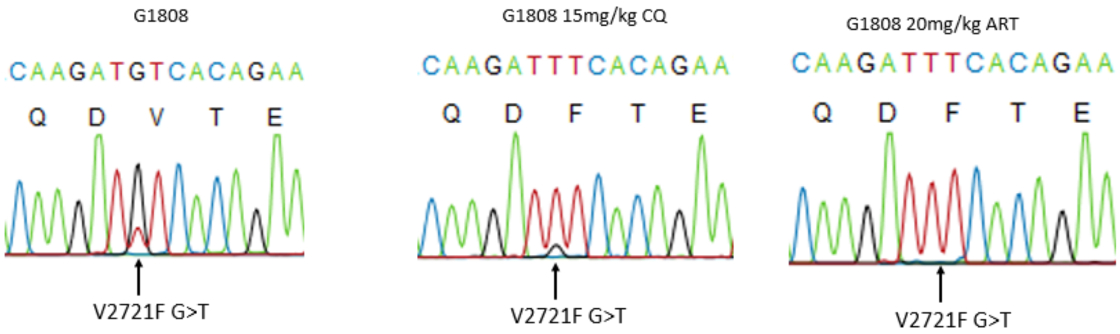

Supplementary figure 3

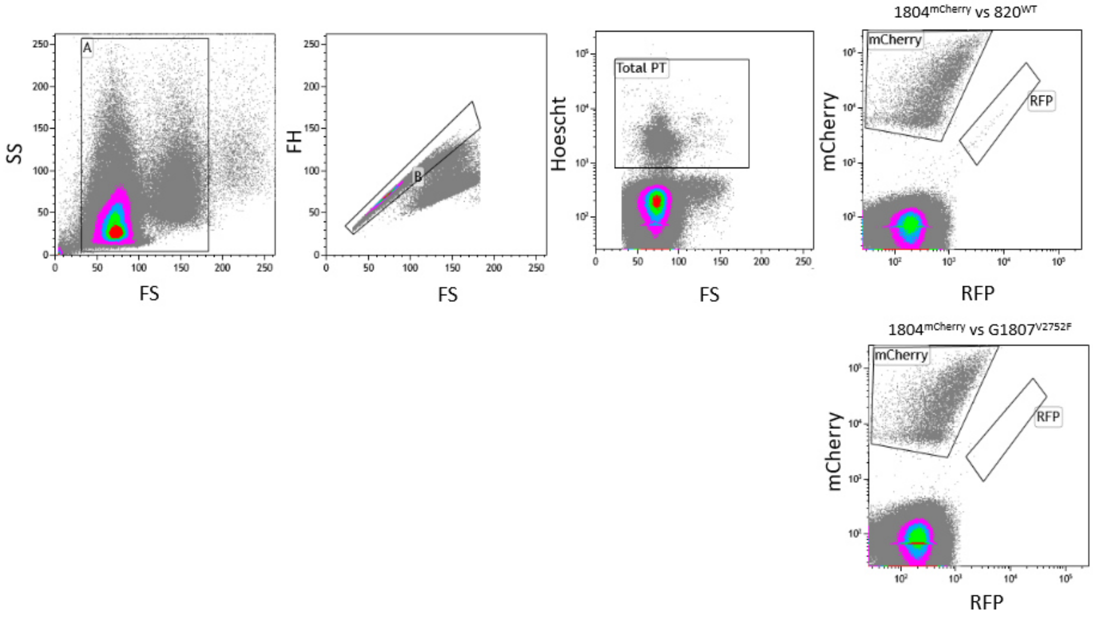

Supplementary figure 4

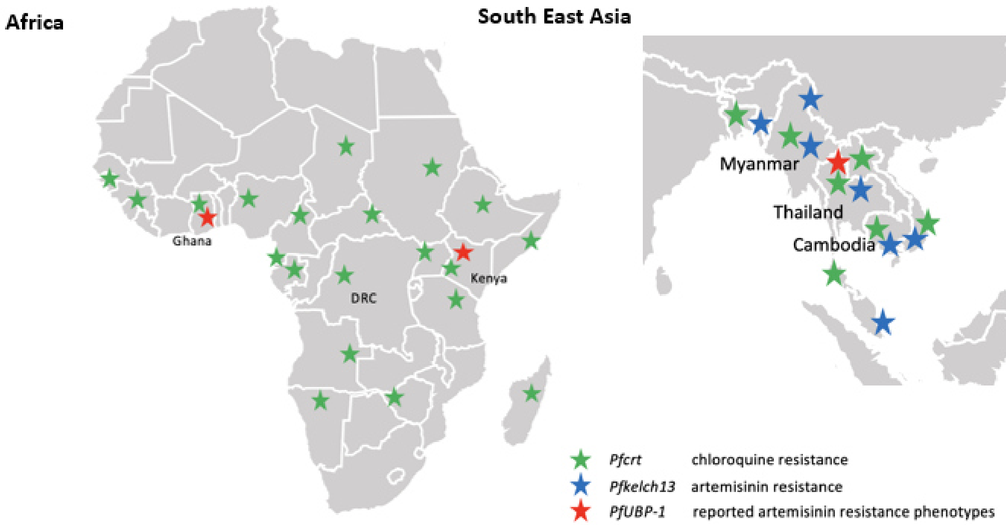

References

1. Ecker A, Lehane AM, Clain J, Fidock DA. 2012. PfCRT and its role in antimalarial drug resistance. Trends Parasitol 28:504-14.
2. Mbengue A, Bhattacharjee S, Pandharkar T, Liu H, Estiu G, Stahelin RV, Rizk SS, Njimoh DL, Ryan Y, Chotivanich K, Nguon C, Ghorbal M, Lopez-Rubio JJ, Pfrender M, Emrich S, Mohandas N, Dondorp AM, Wiest O, Haldar K. 2015. A molecular mechanism of artemisinin resistance in Plasmodium falciparum malaria. Nature 520:683-7.
3. Ashley EA, Dhorda M, Fairhurst RM, Amaratunga C, Lim P, Suon S, Sreng S, Anderson JM, Mao S, Sam B, Sopha C, Chuor CM, Nguon C, Sovannaroth S, Pukrittayakamee S, Jittamala P, Chotivanich K, Chutasmit K, Suchatsoonthorn C, Runcharoen R, Hien TT, Thuy-Nhien NT, Thanh NV, Phu NH, Htut Y, Han K-T, Aye KH, Mokuolu OA, Olaosebikan RR, Folaranmi OO, Mayxay M, Khanthavong M, Hongvanthong B, Newton PN, Onyamboko MA, Fanello CI, Tshefu AK, Mishra N, Valecha N, Phyo AP, Nosten F, Yi P, Tripura R, Borrmann S, Bashraheil M, Peshu J, Faiz MA, Ghose A, Hossain MA, Samad R, et al. 2014. Spread of artemisinin resistance in Plasmodium falciparum malaria. The New England journal of medicine 371:411-423.
4. Menard D, Khim N, Beghain J, Adegnika AA, Shafiul-Alam M, Amodu O, Rahim-Awab G, Barnadas C, Berry A, Boum Y, Bustos MD, Cao J, Chen JH, Collet L, Cui L, Thakur GD, Dieye A, Djalle D, Dorkenoo MA, Eboumbou-Moukoko CE, Espino FE, Fandeur T, Ferreira-da-Cruz MF, Fola AA, Fuehrer HP, Hassan AM, Herrera S, Hongvanthong B, Houze S, Ibrahim ML, Jahirul-Karim M, Jiang L, Kano S, Ali-Khan W, Khanthavong M, Kremsner PG, Lacerda M, Leang R, Leelawong M, Li M, Lin K, Mazarati JB, Menard S, Morlais I, Muhindo-Mavoko H, Musset L, Na-Bangchang K, Nambozi M, Niare K, Noedl H, et al. 2016. A Worldwide Map of Plasmodium falciparum K13-Propeller Polymorphisms. N Engl J Med 374:2453-64.
5. Cerqueira GC, Cheeseman IH, Schaffner SF, Nair S, McDew-White M, Phyo AP, Ashley EA, Melnikov A, Rogov P, Birren BW, Nosten F, Anderson TJC, Neafsey DE. 2017. Longitudinal genomic surveillance of Plasmodium falciparum malaria parasites reveals complex genomic architecture of emerging artemisinin resistance. Genome biology 18:78-78.
6. Adams T, Ennuson NAA, Quashie NB, Futagbi G, Matrevi S, Hagan OCK, Abuaku B, Koram KA, Duah NO. 2018. Prevalence of Plasmodium falciparum delayed clearance associated polymorphisms in adaptor protein complex 2 mu subunit (pfap2mu) and ubiquitin specific protease 1 (pfubp1) genes in Ghanaian isolates. Parasites & vectors 11:175-175.
7. Henriques G, Hallett RL, Beshir KB, Gadalla NB, Johnson RE, Burrow R, van Schalkwyk DA, Sawa P, Omar SA, Clark TG, Bousema T, Sutherland CJ. 2014. Directional selection at the pfmdr1, pfcrt, pfubp1, and pfap2mu loci of Plasmodium falciparum in Kenyan children treated with ACT. J Infect Dis 210:2001-8.
8. Borrmann S, Straimer J, Mwai L, Abdi A, Rippert A, Okombo J, Muriithi S, Sasi P, Kortok MM, Lowe B, Campino S, Assefa S, Auburn S, Manske M, Maslen G, Peshu N, Kwiatkowski DP, Marsh K, Nzila A, Clark TG. 2013. Genome-wide screen identifies new candidate genes associated with artemisinin susceptibility in Plasmodium falciparum in Kenya. Scientific reports 3:3318-3318.
